# The leishmanicidal effect of *Lucilia sericata* larval saliva and hemolymph on* in vitro Leishmania tropica*

**DOI:** 10.1186/s13071-020-04543-y

**Published:** 2021-01-11

**Authors:** Sara Rahimi, Ali khamesipour, Amir Ahmad Akhavan, Javad Rafinejad, Reza Ahmadkhaniha, Mahmood Bakhtiyari, Arshad Veysi, Kamran Akbarzadeh

**Affiliations:** 1 Department of Medical Entomology and Vector Control, School of Public Health, Tehran University of Medical Sciences, Tehran, Iran; 2 Center for Research and Training in Skin Diseases and Leprosy, Tehran University of Medical Sciences, Tehran, Iran; 3 Pharmaceutical Chemistry, Department of Human Ecology, School of Public Health, Tehran University of Medical Sciences, Tehran, Iran; 4 Department of Community Medicine and Epidemiology, School of Medicine Non-communicable Diseases Research Center Alborz, University of Medical Sciences, Karaj, Iran; 5 Zoonoses Research Center, Research Institute for Health Development, Kurdistan University of Medical sciences, Sanandaj, Iran

**Keywords:** Anti-leishmanial activity, Saliva, *Leishmania tropica*, Promastigote, Intracellular amastigote, Hemolymph

## Abstract

**Background:**

Leishmaniasis is a major parasitic disease worldwide, except in Australia and Antarctica, and it poses a significant public health problem. Due to the absence of safe and effective vaccines and drugs, researchers have begun an extensive search for new drugs. The aim of the current study was to investigate the *in vitro* leishmanicidal activity of larval saliva and hemolymph of *Lucilia sericata* on *Leishmania tropica*.

**Methods:**

The effects of different concentrations of larval products on promastigotes and intracellular amastigotes of *L. tropica* were investigated using the mouse cell line J774A.1 and peritoneal macrophages as host cells. The 3-(4.5-dimethylthiazol-2-yl)-2,5-diphenyltetrazolium bromide (MTT) assay and direct observation and counting method were used to assess the inhibitory effects and cell cytotoxicity of the larval products. The effects of larval products on the amastigote form of *L. tropica* were quantitatively estimated by calculating the rate of macrophage infection, number of amastigotes per infected macrophage cell, parasite load and survival index.

**Results:**

The 50% cytotoxicity concentration (CC_50_) value of both larval saliva and hemolymph was 750 µg/ml, and the 50% inhibitory concentration (IC_50_) values were 134 µg/ml and 60 µg/ml for larval saliva and larval hemolymph, respectively. The IC_50_ for Glucantime, used a positive control, was (11.65 µg/ml). Statistically significant differences in viability percentages of promastigotes were observed for different doses of both larval saliva and hemolymph when compared with the negative control (*p* ≤ 0.0001). Microscopic evaluation of the amastigote forms revealed that treatment with 150 µg/ml larval hemolymph and 450 µg/ml larval saliva significantly decreased the rate of macrophage infection and the number of amastigotes per infected macrophage cell.

**Conclusion:**

Larval saliva and hemolymph of *L. sericata* have acceptable leishmanicidal properties against *L. tropica*.

**Graphical Abstract:**

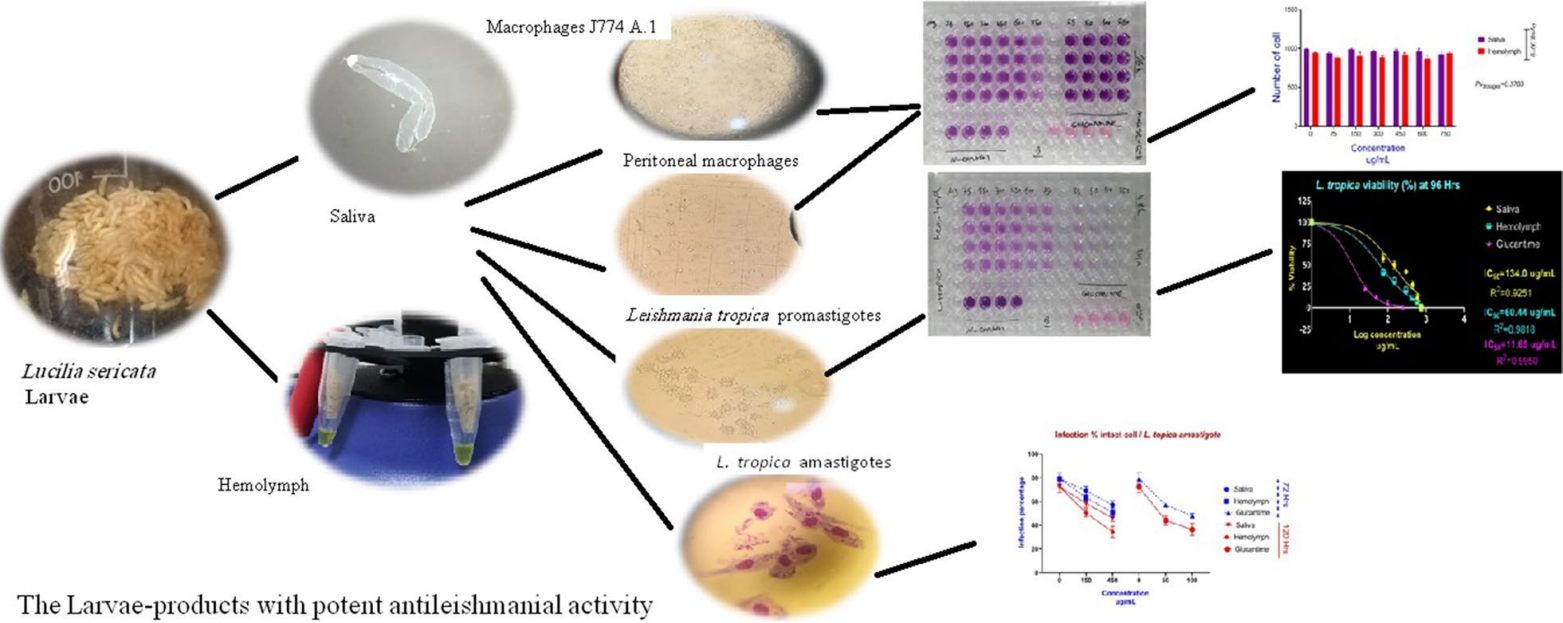

## Introduction

Leishmaniasis is a vector-borne disease caused by 20 pathogenic *Leishmania* species and transmitted to humans by about 30 species of female phlebotominae sand flies [1-3]. The World Health Organization (WHO) considers leishmaniasis as 1 of the 7 important infectious diseases and among the 15 most Neglected Tropical Diseases in the world [1, 3]. Leishmaniasis is endemic in more than 100 countries across four continents. It is estimated that 700,000 to 1.2 million new cases are recorded every year, and about 350 million people are at risk of contracting the infectious disease [2-4].

Clinical manifestations vary from self-healing cutaneous (cutaneous leishmaniasis) lesions to fatal visceral (visceral leishmaniasis) disease depending on the interaction between the *Leishmania* species and host’s immune response [3, 5]. Cutaneous leishmaniasis (CL) is widely distributed in Afghanistan, Algeria, Brazil, Colombia, Costa Rica, Ethiopia, Peru, Sudan, the Syrian Arab Republic and the Islamic Republic of Iran, where 0.7 million new cases are reported annually [1, 2, 6]. In Iran, *Leishmania tropica* is the main agent of anthroponotic cutaneous Leishmaniasis (ACL). The ACL form of the disease is endemic in 14 foci in 8 provinces, including Tehran in the central part of the country, Mashhad, Neishabur and Sabzvar in the northeast, Shiraz in the south and Kerman and Bam in the southeast [7-9]. The main vector of *L. tropica* is *Phlebotomus sergenti*, and the main reservoir host is human but dogs have a role as animal reservoir host, and active lesions in dogs have been reported in Tehran, Mashhad, Shiraz and Neishabur [10].

Due to the toxicity of the current anti-leishmanial drugs, including the antimonials (first-line medication), amphotericin B (AmB) (2nd line medication), imidazoles, miltefosine, paromomycin and liposomal amphotericin B, as well as the emerging resistance, high cost of medications and long duration of the treatment regimen, there have been calls for the development of novel, effective drugs [11, 12].

Natural chemical compounds have been suggested as promising candidates for the development of safe and effective drugs against the disease. A large body of evidence has indicated the potential use of some insect products as drug candidates for the treatment of human diseases. The use of insect products as medicines for the treatment of diseases is termed “bugs as drugs” [13, 14].

Maggot therapy has been widely used for the treatment of various kinds of wounds. One of the widely used larvae for maggot therapy is *Lucilia sericata* [15-17]. The mechanisms of action of maggot therapy involve the removal of necrotic tissues and stimulation of tissue granulation by the larvae. The larvae also exhibit antiseptic effects [18, 19]. Anti-microbial peptides (AMPs) released into the wound through larval excretion and secretions (ES) have microbicidal effects against pathogenic microorganisms such as gram-positive and -negative bacteria [20-24], fungi [25, 26] and parasites [15, 27, 28]. Among the 800 AMPs that have been isolated from natural sources (Antimicrobial Peptide Database, http://aps.unmc.edu/AP/main.php), approximately half have been derived from insects [14].

Previous studies have investigated the effectiveness of the larval ES in the treatment of CL induced in murine and hamster models using *Leishmania amazonesis* [29] and in a *L. major*-infected murine model [28]. Also, the effectiveness of the larval ES in *in vitro* CL models infected with *L. tropica* [30] and *in vivo* models infected with *Leishmania panamensis* [15] has been investigated.

The aim of the present study was to evaluate the anti-*Leishmania* effects of larval saliva and hemolymph of *L. sericata* against *L. tropica*. Also, we analyzed and compared the effectiveness of salivary gland lysate (SGL) of laboratory-bred larvae with field-caught larvae against *L tropica*. In the present study, the activity of larval products of *L. sericata* on *L. tropica* promastigotes and intracellular parasites and the cytotoxic activity of larval hemolymph and SGL against the murine macrophage cell line, J774A.1 cells and peritoneal macrophages were evaluated by cell viability assays, and the efficacies of the larval products were compared with Glucantime.

## Materials and methods

### Adult fly collection

The adult flies were caught by hand collection and bait trap methods from different habitats including livestock farms, gardens and slaughterhouses in Tehran County between April–June 2018. Adults were transferred to the insectary of Cyclorrhapha flies at the School of Public Health, Tehran University of Medical Sciences (SPH-TUMS).

In this study, adult flies were anesthetized by cold shock and were then subjected to morphological identification using morphological keys [31]. The field strain of *L. sericata* was reared in a separate place at the insectary to obtain first-generation (F1) larvae. The F1 larvae were reared to the third-instar stage, and their SGL protein profiles were compared with third-instar laboratory-bred larvae using the SDS-PAGE technique. The adult flies were fed with beef liver and meat, which is their preferred medium for egg laying.

### Fly rearing

A laboratory-bred strain of *L. sericata* was obtained from colonies maintained at the insectary of Cyclorrhapha flies at the SPH-TUMS. These colonies have been grown and maintained in the laboratory since December 2012.

Adult flies were kept and maintained in 46 × 46 × 46 cm cages under controlled conditions in a rearing room at 27 ± 3 °C, 45 ± 5% relative humidity and 16:8 h light/dark [32]. Adult flies were offered granulated sugar, water and palm dates as well as a piece of beef liver to provide the essential carbohydrates and proteins and suitable oviposition surface.

Laid egg batches were transferred to a rearing jar, and newly hatched larvae were fed on beef liver daily. Under the above-mentioned conditions, third-instar larvae appeared on the 3rd day. A 12-mm-long third-instar larva was used as the standard size for all experiments [33].

### Sterile larvae preparation

Larvae were collected from the rearing jars, washed in sterile distilled water, immersed in 4% deconex and then rinsed three times with sterile distilled water. Finally, the larval specimens were sterilized in 70% isopropyl alcohol (IPA) and dried on a sterilized napkin. Laboratory tests for bacterial infection were performed before preparation of larva SGLs and hemolymph.

### Preparation of salivary gland lysate (SGLs)

The salivary glands of the third-instar larvae were dissected and transferred into cold fresh phosphate-buffered saline (PBS), pH 7.2. The salivary gland tissues were then stored at – 20 ºC until use. Before use, salivary glands were disrupted by three cycles of freeze/thaw in liquid nitrogen and boiling water for a few seconds. After the centrifugation of the homogenate at 18,000*g* for 15 min, the SGL supernatants were used for subsequent tests [7, 34].

### Protein measurement

The concentration of SGL proteins was determined by the BCA Protein Assay Kit (Takara Biotechnology, no. T9300A, Japan), following the manufacturer’s instructions. Standard proteins were prepared from bovine serum albumin (BSA) in sodium azide.

### Preparation of larval hemolymph

The anterior part of the third-instar larvae (near the mouth hooks) was cut using small scissors. Each 0.5-ml micro-tube was cut about 3–4 mm straight down the center using a razor blade, and batches of ten larvae were put in 0.5-ml micro-tubes. Finally, each of the prepared 0.5-ml tubes was placed in a larger 1.5-ml micro-tube for centrifugation for 5–10 s to isolate the hemolymph from the larvae. This step was performed immediately before use of the hemolymph to prevent melanization of the hemolymph product.

### SDS-PAGE

Larval SGLs of *L. sericata* were extracted, and the proteins and/or glycoproteins were visualized by SDS-PAGE on 1-mm-thick 12.5% Tris-glycine gel with 110-V fixed voltage using “Mini-Protein III” (Bio-Rad, Munich, Germany) under reducing conditions.

The SGLs from three to five pooled glands were loaded into each well. Following electrophoresis, the gels were stained with silver nitrate according to the methods described by Heukeshoven and Dernick [35]. A pre-stained protein ladder (PageRuler, Fermentas) was used to estimate the molecular weights of the protein bands.

### Parasite culture

Promastigotes of *L. tropica* (MHOM/IR/01/YAZA) were obtained from patients referred to the Center for Research and Training in Skin Diseases and Leprosy (CRTSDL) of TUMS for treatment of CL infection. The amastigotes were grown in Novy-Macneal-Nicolle (NNN) medium and sub-cultured in RPMI 1640 supplemented with 10% heat-inactivated fetal bovine serum (FBS), penicillin and streptomycin (100 μg/ml) at 26 ± 1 °C.

### Culture of the murine macrophage cell line, J774A.1 cells

The J774A.1 cells were obtained from the National Cell Bank of Iran (Pasteur Institute, Tehran, Iran) and cultured in DMEM medium supplemented with 15% FBS, penicillin and streptomycin (100 μg/ml) at 37 °C in a 5% CO_2_ humidified incubator.

### Culture of murine peritoneal macrophages

Peritoneal cells were collected from the peritoneal cavity of 4–5-week-old female BALB/c mice. For this purpose, each mouse was injected intraperitoneally (IP) with 3 ml of sterile 3% thioglycollate medium and then anesthetized by IP injection of sodium pentobarbital (100 mg/kg) after 72 h.

The anesthetized mice were killed by cervical dislocation. The whole body of each mouse was washed using 70% ethanol; 5 ml cold PBS was injected into the peritoneal cavity of each mouse. Following PBS injection, the peritoneal cells were aspirated. The cell suspension was centrifuged for 10 min at 400*g* in a refrigerated centrifuge and resuspended in RPMI 1640 supplemented with 15 % FBS. The cells were then incubated at 37 °C in a 5% CO_2_ incubator. After incubation, the cells were counted, and the viability of the cells was assessed.

### *Leishmania tropica* promastigote susceptibility to larval saliva and hemolymph of *L. sericata*

Promastigotes of *L. tropica* were harvested at log-phase, and 1 ×10^5^ parasites per well were cultured on a 96-well plate using complete RPMI 1640 medium. Then, larval saliva or hemolymph was added at 75, 150, 300, 450, 600 and 750 µg/ml concentrations and incubated at 26 ± 1 °C for 24, 48, 72 and 96 h.

The number of viable parasites was determined by direct observation under the light microscope and 3-(4.5-dimethylthiazol-2-yl)-2,5-diphenyltetrazolium bromide (MTT) assay. Briefly, MTT (0.5 mg/ml) was dissolved in saline solution, and the solution was further sterilized using 0.22-mm filters.

About 200 ul of MTT solution was added to each well and incubated at 26 ± 1 °C for an additional 4 h. The supernatant was gently removed, and the formazan crystals were solubilized by 100 μl dimethyl sulfoxide (DMSO). The optical density (OD) of the plates was determined using the ELISA reader (Bio-Tek ELX 808 iu) at 570/630 nm [36].

Different concentrations of Glucantime (25, 50,100, 250 µg/ml) were used as positive controls and sterile PBS were used as a negative control. All the experiments were performed in quadruplicate, and the results were compared with the negative and positive controls.

### Cytotoxicity test for larval products against peritoneal macrophages and murine macrophage cell line, J774 cells

The peritoneal macrophages and J774 cells were plated at 1 × 10^5^ and 5 × 10^4^ cells per well, respectively, on a 96-well culture plate in complete RPMI 1640 medium at 37 °C and 5% CO_2_ condition. Following bonding time, the larval products were added at concentrations of 75, 150, 300, 450, 600 and 750 µg/ml. Cells with and without treatment were incubated at 37 °C for 24, 48, 72, 96 and 120 h.

The number of viable cells (cell viability assay) was determined by direct observation trypan blue test and 3-(4.5-dimethylthiazol-2-yl)-2,5-diphenyltetrazolium bromide (MTT) assay. The trypan blue dye exclusion test distinguishes dead cells from viable cells by color change. The MTT assay was similar to what we described in the parasite viability assay.

The values of 50% cytotoxicity concentration (CC_50_) and 50% inhibitory concentration (IC_50_) were calculated by non-linear regression tests, and the selectivity index (SI) was determined by the CC_50_/IC_50_ ratio [37].

### Amastigote susceptibility to larval saliva and hemolymph of *L. sericata*

The peritoneal macrophages and J774 cells were dispensed in an eight-well chamber slide at 7 × 10^4^ and 2 × 10^4^ cells/well, respectively, and the samples were incubated at 37 °C, 5% CO_2_ for 6 h to allow the adherence of cells.

The adherent cells were washed with warm RPMI medium, then infected with stationary-phase *L. tropica* promastigote at 10:1 parasite/cell and incubated again in complete RPMI medium. After 24 h, the infected cells were gently washed with warm RPMI medium to remove non-internalized promastigotes and were then treated with larval products at a concentration of 150 and 450 µg/ml in triplicate for 72 and 120 h.

Glucantime was used as positive control (standard drug), and macrophages containing amastigotes without treatment were used as negative controls.

Finally, each slide was dried, fixed and stained with Giemsa staining. The stained slides were examined under light microscope. The percentage of infected macrophages and mean number of amastigotes per 100 macrophages were calculated and compared with the untreated control group.

### Statistical analysis

Continuous baseline demographic and *in vitro* data have been presented as mean ± standard deviation (SD) and grouped data as frequencies and percentages. Departure from normality assumption was assessed by the Kolmogorov-Smirnov test.

Chi-square and/or Fisher’s exact tests were used to determine the independence of two categorical variables.

A paired *T*-test was used to evaluate the differences in mean within each treatment group. The utilized assumptions of the parametric statistics were conformed to the data by checking the data normality.

One-way ANOVA followed by Bonferroni multiple comparison tests was employed to investigate the differences in mean between the different groups. To investigate the differences between parasite and macrophage cell fatality under different treatments, *t* concentrations and time points, we used the generalized estimation equation (GEE) method developed by Liang and Zeger.

The GEE is a widely used estimation method for marginal (i.e. population-averaged) modeling of repeated data. In brief, GEEs use the generalized linear model to estimate more efficient and unbiased regression parameters relative to ordinary least square regression in part, because they permit specification of a working correlation matrix that accounts for the form of within-subject correlation of responses on dependent variables of many different distributions, including normal, binomial, and Poisson.

The GraphPad Prism’s (8.0.2) dose-response (variable slope) equation [log (inhibitor) *vs* normalized response] was used to estimate the CC50 and IC50 values of the larval products against both of the macrophage cell types and *L. tropica* promastigotes.

Infection rate (%*I*), decrease in infection rate (%DI), viability percentage of amastigotes (%*V*), percent decrease in viability of amastigotes (%DV), parasite load, survival index and selectivity index were defined for analyzing the amastigote and promastigote susceptibility to the larval products and toxicity of the larval products to the macrophage cell types (Table 1).

**Table 1:** Parameters for evaluating amastigote susceptibility to larval-derived products

Parameter	Abbreviation	Equation
Infection percentage	*I* %	(# Infected cells/100 randomly chosen cells) × 100
Decreased in infection percentage	DI %	[(%*I* no treatment − %*I* treatment)/%*I* no treatment] × 100
Viability of amastigote percentage	*V* %	(# amastigote treatment/# amastigote no treatment) × 100
Decreased viability of amastigote percentage	DV %	[(# amastigote no treatment − (# amastigote treatment/no treatment] × 100
Parasite load	PL	# amastigotes/# infected cells
Survival index	SVI	%*I* × PL
Selectivity index	SI	CC_50_/IC_50_

STATA version 13 MP was used to perform all the statistical analyses, and *p* values ≤ 0.05 were considered statistically significant.

## Results

### Protein concentration of larval salivary gland and hemolymph of *L. sericata*

The average protein contents of one pair of larval salivary glands were 5.7 and 14.7 µg for field- and laboratory-bred *L. sericata* larvae, respectively. The average protein contents of larval hemolymph isolated from *L. sericata* specimens were 213 and 314 µg for field- and laboratory-bred larvae, respectively.

### Salivary gland protein profiles *of the Lucilia sericata* larvae

**Fig. 1:**
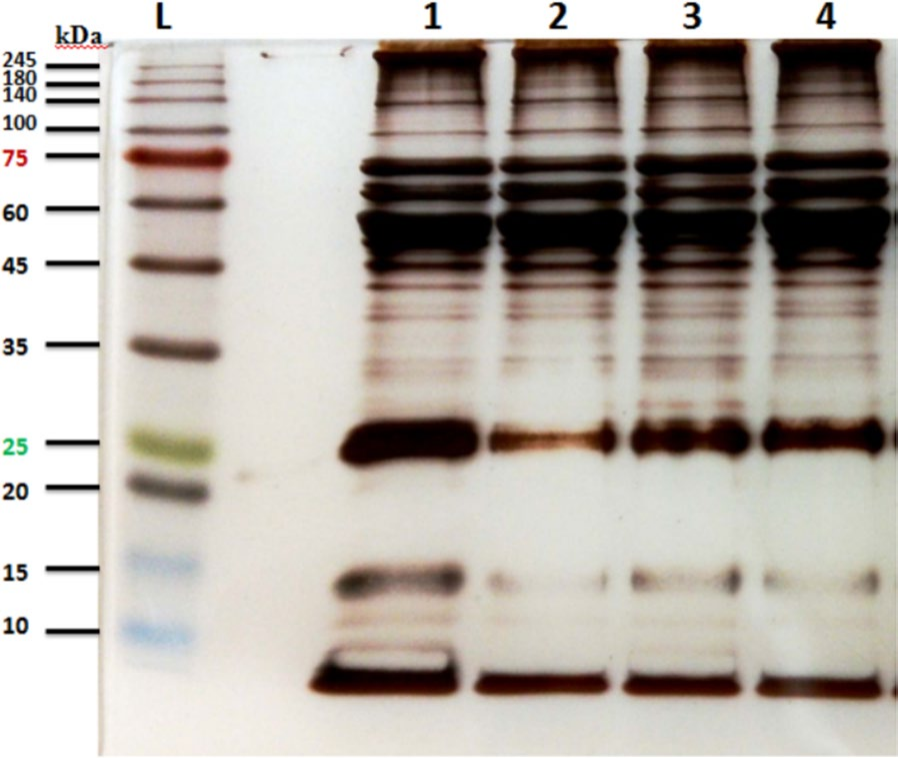
SDS-PAGE analyses of salivary gland lysates of field- and laboratory-bred *L. sericata* third-instar larvae. L: pre-stained protein ladder (page-ruler); lanes 1 and 2: laboratory-bred larvae; lanes 3 and 4: field larvae collected from Tehran Province, Iran

About 17–19 protein bands were observed in the 12.5% polyacrylamide gel, with molecular weight of 10–245 kDa. The electrophoretic protein patterns of SGL proteins of field- and laboratory-bred *L. sericata* larvae are shown in Fig. 1.

The SGL proteins of field- and laboratory-bred larvae were separated into ten major protein bands with molecular masses of 15–140 kDa and 8 faint bands of about 12–15 and 27–42 kDa in both field- and laboratory-bred larvae. Interestingly, the SGL profiles of field third-instar larvae were completely similar to those of the laboratory-bred third-instar larvae.

### *Leishmania tropica *promastigote susceptibility to larval products of *L. sericata*

The IC_50_ values of larval saliva and hemolymph of *Lucilia sericata* were evaluated against parasite promastigotes at 24, 48, 72 and 96 h.

**Fig. 2:**
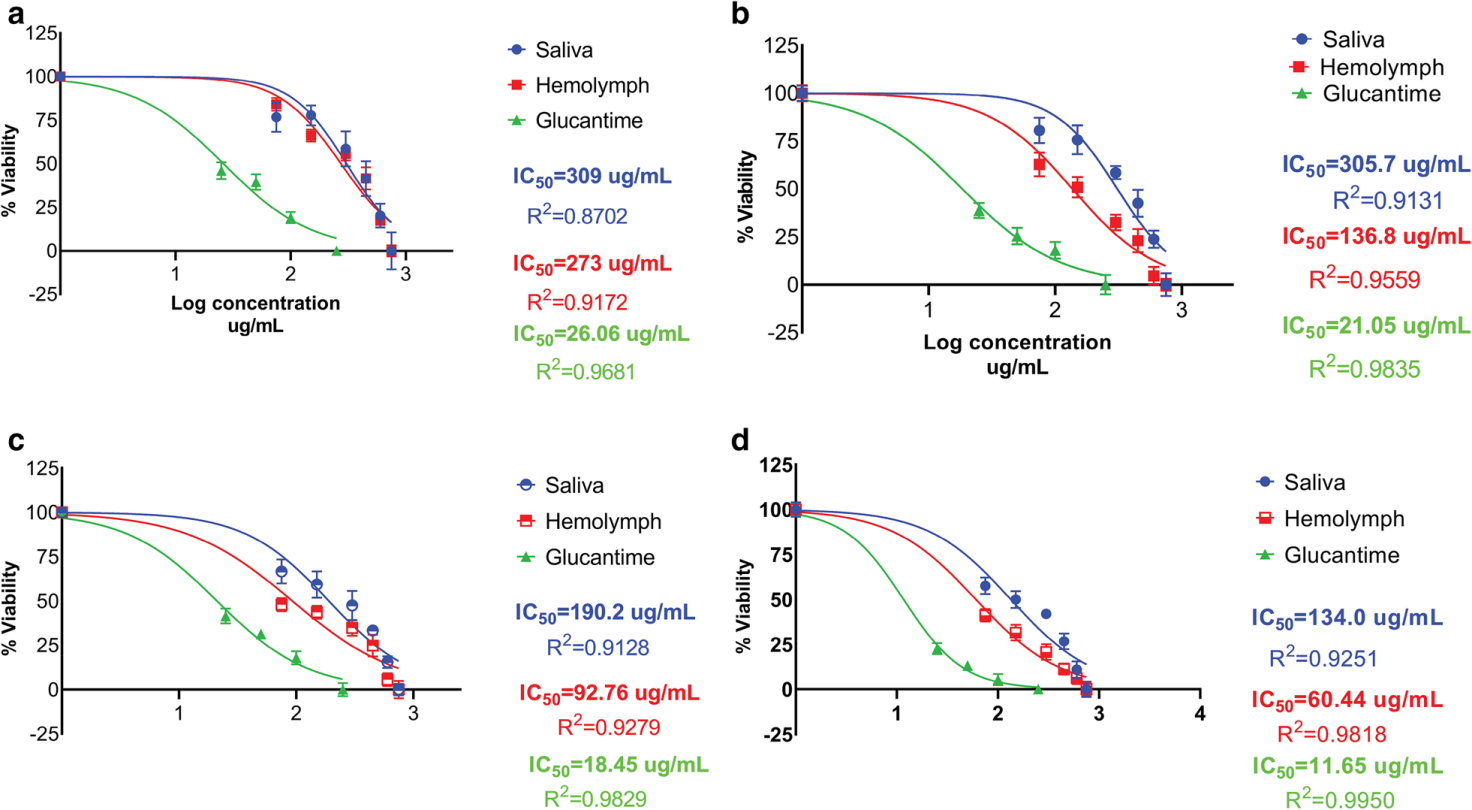
Dose-response curves regarding the effect of *Lucilia sericata* larval-derived products on *Leishmania tropica* promastigotes (IC_50_). **a** Larval saliva IC_50_ *vs* larval hemolymph IC_50_ at 24 h. **b** Larval saliva IC_50_ *vs* larval hemolymph IC_50_ at 48 h. **c** Larval saliva IC_50_ *vs* larval hemolymph IC_50_ at 72 h. **d** Larval saliva IC_50_ *vs* larval hemolymph IC_50_ at 96 h and compared with standard drug

The lowest IC_50_ values were 134.0 µg/ml (log = 2.127) and 60.44 µg/ml (log =  1.781) at 96 h for the larval saliva and hemolymph, respectively. The results were compared with those of Glucantime (Fig. 2).

Statistically significant differences in the viability percentage of promastigotes were observed for both larval saliva and hemolymph at different doses compared with the negative control (*p* ≤ 0.0001). Also, there was a statistically significant difference in viability percentage of promastigotes treated with saliva compared with Glucantime (*p* = 0.0001), but the difference was not significant between hemolymph and Glucantime (*p* = 0.806).

MTT assay showed strong toxicity of the larval products against promastigotes, which increased with an increase in concentration. The lowest viability percentage was 24% for promastigotes treated with saliva and 11% for promastigotes treated with hemolymph. However, treatment with very high concentrations of larval products for 120 h had no toxic effect on both types of macrophages, with cell viability of both types of macrophages > 95%.

**Fig. 3:**
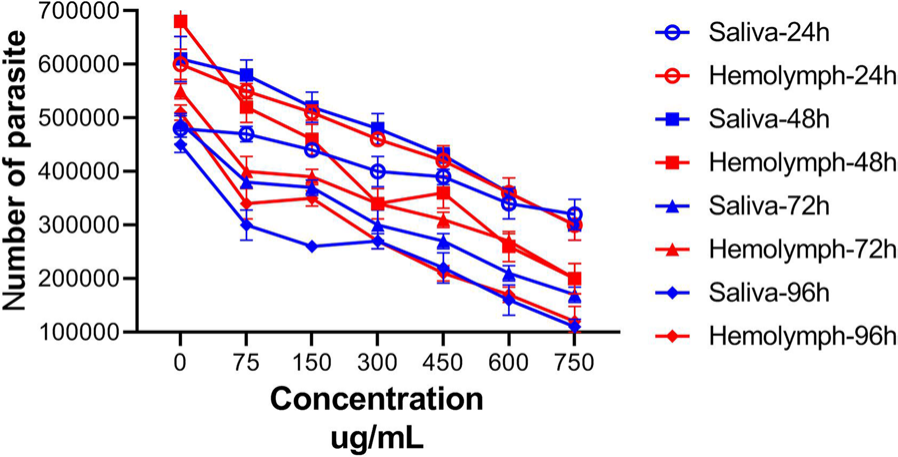
The number of alive promastigotes exposed to larval-derived products in different dosages at different time points

Figure 3 illustrates the number of live parasite promastigotes observed directly under the light microscope. The selectivity indexes (SI) of *L. sericata* larval products are showed in Table 2.

**Table 2:** Selectivity index for *Leishmania tropica* promastigote treatment with saliva and hemolymph at different time points

Treatment	Selectivity index (CC_50_/IC_50_)			
Time:	24 h	48 h	72 h	96 h
Saliva	2.38	2.42	3.94	5.59
Hemolymph	2.74	5.48	8.08	12.4

### Cytotoxicity of larval products of *Lucilia sericata* to peritoneal macrophages and murine macrophage cell line and J774 cells

**Fig. 4:**
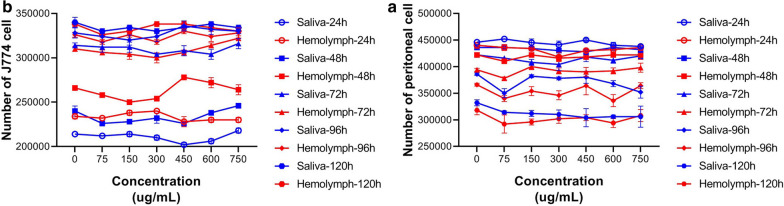
The number of viable cells treated with larval-derived products by trypan blue in different dosages at different time points. **a** The number of peritoneal viable cells treated with larva-derived products in different concentrations at different time points. **b** The number of J774 viable cells treated with larva-derived products in different concentrations at different time points

The number of viable cells was obtained for both types of macrophages at 24, 48, 72, 96 and 120 h (Fig. 4). The viability percentage of macrophages was estimated by using the MTT assay at the time points specified above after exposure to larval saliva and hemolymph.

MTT assays showed that larval saliva and hemolymph did not have any significant toxic effect on either mouse peritoneal macrophages or J774 cells in different doses and time points. There were no significant differences in the viability percentage of both macrophage types when treatments with larval saliva, hemolymph and Glucantime were compared (*p* = 0.439). However, there was a significant difference in cell viability percentage between the two types of macrophages (*p* = 0.0001) after treatment with all three treatments.

### Amastigote susceptibility to larval saliva and hemolymph of *L. sericata*

**Fig. 5:**
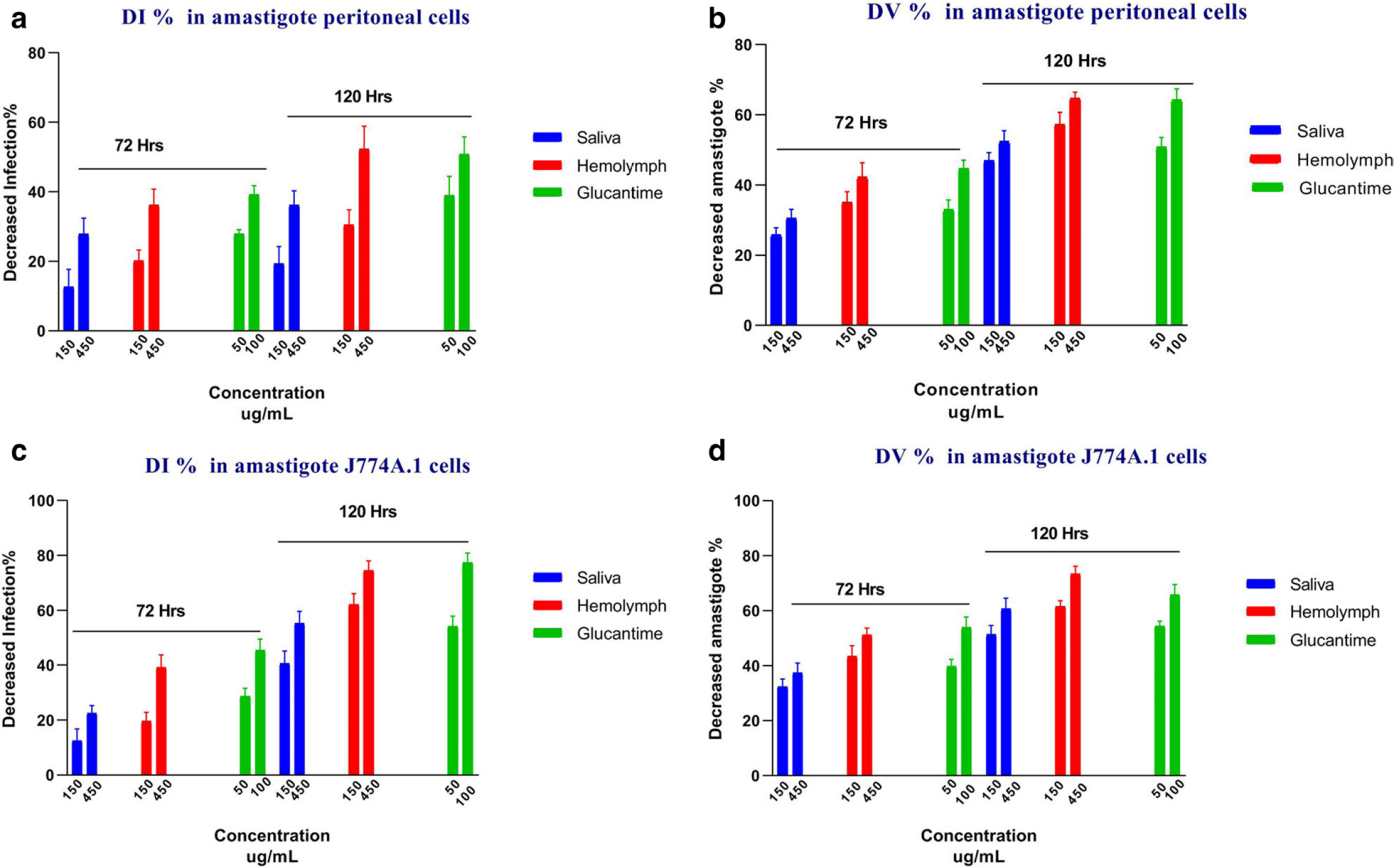
*Leishmania tropica* amastigote susceptibility to *Lucilia sericata* larval products at 72 and 120 h compared with Glucantime. **a** Decrease in infection percentage (DI %) in *L. tropica* amastigote peritoneal cells. **b** Decrease in amastigote viability (DV %) in *L. tropica* amastigote peritoneal cells. **c** Decrease in infection percentage (DI %) in *L. tropica* amastigote J774A.1 cells. **d** Decrease in amastigote viability (DV %) in *L .tropica* amastigote J774A.1 cells

The infection rate (*I*%), viability percentage of amastigotes (*V*%), decrease in infection rate (DI%) and percent decrease in amastigote viability (DV%) of both treatments with saliva and hemolymph are summarized in Table 3 and Fig. 5.

**Table 3:** Effect of *Lucilia sericata* larval-derived products on *Leishmania tropica* amastigote in *in vitro* conditions

Treatment	Dosages (µg/ml)	Amastigote *L. tropica*/peritoneal cell				Amastigote *L. tropica*/J774 cell line			
		*I*%		*V*%		*I*%		*V*%	
Time (h)		72 h	120 h	72 h	120 h	72 h	120 h	72 h	120 h
No treatment control	0	79.3 ± 4.7	72.3 ± 4.7	100 ± 0	100 ± 0	66.3 ± 4	59 ± 3.6	100 ± 0	100 ± 0
Saliva	150	69 ± 4	58.3 ± 3.5	74.2 ± 2	53.1 ± 2.3	58 ± 4	35 ± 2.6	67.5 ± 3.2	48.7 ± 3.3
	450	57 ± 3.6	46 ± 3	69.5 ± 2.7	47.7 ± 3.1	51.3 ± 1.5	26.3 ± 2.5	62.4 ± 3.2	39 ± 3.8
Hemolymph	150	63.3 ± 2.5	50.3 ± 3.2	64.5 ± 3	42.8 ± 3.4	56 ± 4.4	22 ± 4	56.6 ± 3.9	38.4 ± 2
	450	50.7 ± 4	34.3 ± 4.7	57.7 ± 4	35.9 ± 2.8	40.3 ± 3.1	15 ± 3	48.7 ± 2.6	26.6 ± 2.8
Positive control	50	57 ± 1	44 ± 4	66.9 ± 2.7	49.3 ± 2.8	47.3 ± 3.8	27.3 ± 2.5	60.3 ± 2.7	45.7 ± 1.9
Glucantime	100	48 ± 2	36.3 ± 5.1	55.4 ± 2.5	35.7 ± 3.1	36 ± 2.6	13.3 ± 3.2	46.2 ± 4	34 ± 3.3

*I*%: infection percentage; *V*%: viability of amastigote

The infection rate and percentage of viable amastigotes significantly decreased after treatment with larval products for 72 and 120 h compared to the control group (*p* = 0.0001). A statistically significant difference in the rate of macrophage infection and the number of amastigotes per infected macrophage cell was observed between treatment with saliva and Glucantime (*p* = 0.050), but the difference was not significant between treatment with hemolymph and Glucantime (*p* = 0.880).

Based on this result, treatment with *L. sericata* larval products decreased *I*% and *V*% at higher concentrations and time points (*p *= 0.003) (Table 3). In other words, treatment with higher concentrations of larval products and at higher time points increased the DI% and DV% values (Fig. 5). There were no statistically significant differences in DI% and DV% between treatment with hemolymph at 150 or 450 µg/ml and Glucantime at 50 µg/ml (*p* = 0.321) as well as treatment with hemolymph at 450 µg/ml and Glucantime at 100 µg/ml (*p* = 0.408).

The average parasite load was 2.12 ± 0.56 amastigotes/peritoneal cell (ama/type of cell) when the macrophages were treated with 100 µg/ml of glucantime (positive control) for 72 h. The average parasite load decreased to 1.77 ± 0.34 after 120 h. For the negative control group, the parasite load was 2.70 ± 0.70 and 3.05 ± 0.57 ama/peritoneal cell at 72 and 120 h, respectively. Treatment with 450 µg/ml of hemolymph resulted in 1.58 ± 0.97 ama/peritoneal cell and 1.20 ± 0.17 ama/J774 cell at 120 h, which were lower compared with the standard drug (Tables 4, 5).

**Table 4:** Parameters for evaluating amastigote *Leishmania tropica* peritoneal macrophage susceptibility to larval-derived products

Treatment	Dosages (µg/ml)	PL		SVI	
		72 h	120 h	72 h	120 h
No treatment control	0	2.70 ± 0.70	3.05 ± 0.57	213.3 ± 4.70	219.6 ± 4.7
Saliva	150	2.28 ± 0.81	2.0 ± 0.62	157.3 ± 2.81	116 ± 3.5
	450	2.14 ± 0.67	1.81 ± 0.75	121.9 ± 3.6	83.2 ± 3
Hemolymph	150	2.17 ± 0.80	1.86 ± 0.35	136.7 ± 2.5	93 ± 3.2
	450	1.95 ± 0.25	1.58 ± 0.97	97.5 ± 4.2	53.7 ± 4.2
Positive control-Glucantime	50	2.16 ± 0.50	1.86 ± 0.40	123.1 ± 5.7	81.8 ± 4

*PL* parasite load, *SVI* survival index

**Table 5:** Parameters for evaluating amastigote *Leishmania tropica* J774A.1 macrophage susceptibility to larval-derived products

Treatment	Dosages (µg/ml)	PL		SVI	
		72 h	120 h	72 h	120 h
No treatment control	0	2.16 ± 0.31	2.03 ± 0.31	142.6 ± 8	120 ± 10
Saliva	150	2.26 ± 0.47	1.55 ± 0.28	131.0 ± 5	54.2 ± 4
	450	2.22 ± 0.33	1.41 ± 0.50	113.2 ± 4	36.6 ± 3
Hemolymph	150	1.88 ± 0.63	1.38 ± 0.44	99.6 ± 5	30.3 ± 3
	450	1.77 ± 0.51	1.20 ± 0.17	70.8 ± 4	18 ± 2
Positive control-Glucantime	50	2.30 ± 0.39	1.70 ± 0.80	108.1 ± 7	45.9 ± 3
	100	2.18 ± 0.75	1.50 ± 0.62	78.4 ± 6	19.5 ± 5

*PL* parasite load, *SVI* survival index

The survival index for the standard drug treatment group was 63.7 ± 5.1 and 19.5 ± 5 (219.6 ± 4.7 and 120 ± 10 for the negative control) for amastigotes of *L. tropica/*peritoneal cells and J774 cells at 120 h. Treatment with 450 µg/ml hemolymph reduced the SI to 53.7 ± 4.2 and 18 ± 2 compared with the negative control at 120 h (Tables 4, 5).

## Discussion

The current study demonstrated the leishmanicidal effects of larval saliva and hemolymph against promastigotes and amastigotes of *L. tropica*, the causative agent of ACL. This study also evaluated the IC_50_ and CC_50_ of larval saliva and hemolymph of *L. sericata* against peritoneal macrophages and the murine macrophage cell line, J774A.1 cells.

Many studies that evaluated the susceptibility and cytotoxicity of *L. tropica* and macrophages to the larval ES of *L. sericata*. However, to our knowledge, there is no study on the effects of larval saliva and hemolymph of *L. sericata* on *L. tropica* and macrophage cells.

A previous study that investigated *Calliphora vicinia* and *L. sericata* larval ES product on the J774 cell line and *L. major* reported that larval ES product concentrations > 40% were highly toxic to macrophages [28], but in the current study, higher concentrations of larval saliva and hemolymph had no toxic effect on both types of macrophage cells up to 120 h, with cell viability > 95%. On the other hand, larval saliva and hemolymph of *L. sericata* had more toxic effects on *L. tropica* promastigotes.

A similar result was reported by another study which demonstrated a greater lethal effect on promastigotes than macrophage cells [38]. Also, the finding of a previous study supports the present finding that larval products have an inhibitory effect on *L. tropica* promastigote and amastigote forms [30].

In the present study, larval hemolymph was found to be highly toxic to promastigotes compared with larval saliva. The viability percentage of promastigotes was < 15% when treated with hemolymph for 96 h. This finding is also in agreement with a previous study that evaluated hemolymph toxicity on gram-positive and -negative bacteria. The previous study found that *L. sericata* larval hemolymph had a stronger toxic effect against gram-positive and -negative bacteria than whole-body extract [39].

Both *L. sericata* larval products had strong leishmanicidal effects against intracellular amastigotes of *L. tropica*. Treatment with larval products resulted in a decrease in infection rate (*I* %) and parasite load, the effect being even stronger for hemolymph treatment than the standard drug (*p* = 0.880).

The decrease in infection rate was > 50% when treated with 150 µg/ml larval hemolymph and 450 µg/ml larval saliva, but even at these concentrations, no toxic effect was exhibited on both types of macrophages exposed to the larval products. The cell viability of the macrophage cells was > 95% when treated with 450 µg/ml hemolymph.

Based on the results of the present study, larval products, especially hemolymph, had high toxicity against the leishmania parasites at concentrations that are not cytotoxic to macrophage cells, which is in line with the study carried out by Laverde-Paz et al. [27].

The leishmanicidal effect of the larval products against amastigotes even at low concentration is a promising outcome for future *in vivo* model experiments. Analysis of the susceptibility of intracellular forms of *L. tropica* to the larval products showed that treatment with lower concentrations for a longer time period had a more toxic effect on the viability of the parasites. It is worth noting that treatment with higher concentrations of larval products resulted in a decrease in the survival index of the parasites.

This finding is not in agreement with that of previous studies [27, 28], which evaluated the susceptibility of the mouse macrophage cell line (J774 cells) infected with amastigotes of *L. major* and U937 human cell line infected with amastigotes of* L. panamensis* to the larval ES of blowfly species. The difference could be explained by comparing the different components and effective substances in the larval-secretion/excretion (ES) used in the previous study and the larval saliva and hemolymph in the present study.

In the present study, the minimum amastigote viability percentage (*V* %) was 35.9 ± 2.8 when treated with 450 µg/ml larval-hemolymph and 26.6 ± 2.8, which performed similarly to Glucantime 100 µg/ml at 120 h. There were differences in *I*, *V*, DI and DV% between the two types of macrophage cells (*p* = 0.0001). This may be due to the genetic manipulation of the J774 macrophage cells, which might have resulted in gain or loss of function of genes.

In the present study, the survival index was determined along with the infection rate (*I* %). The PL and SVI index values after treatment with hemolymph were lower compared with saliva in both types of macrophage cells infected with *L. tropica*. Also, there was a significant decrease in the PL and SVI index between the larval product treatment groups compared with the no treatment control; furthermore, the leishmanicidal effects of larval products were retained as the duration and concentration increased.

The larval hemolymph was the most selective (SI > 5); when the selectivity index was < 1, the product was more toxic against the macrophage cells than against the parasites [40].

Some previous studies on the leishmanicidal effect of larval products on *Leishmania* parasites in *in vitro* and *in vivo* models have reported potential therapeutic effects of larval ES of *L. sericata* on *L. amazonensis* [29] *L. tropica* [30], *L. major* [28] and *L. panamensis* [15]. Previous studies that evaluated *in vitro* models have concentrated on *Leishmania* sub-genus species [27, 28, 30].

In the present study, the *in vitro* leishmanicidal effect of the larval saliva and hemolymph of *L. sericata* against the promastigote and amastigote forms of *L. tropica* was demonstrated.

## Conclusion

For the first time to our knowledge, the therapeutic effect of the larval saliva and hemolymph of *L. sericata* on the promastigote and amastigote forms of *L. tropica* has been demonstrated in this study. In addition, the toxic effect of these larval products on intact (peritoneal cells) and manipulated (J774A.1 cell line) macrophage cells was investigated.

It is worth mentioning that the larval hemolymph of *L. sericata* had equivalent effectiveness at 450 µg/ml compared with a standard drug (100 µg/ml), and its effect was maintained when the concentration was increased. The larval hemolymph has a strong toxic effect on *L. tropica* at completely safe concentrations for macrophage cells. Accordingly, larval products of *L. sericata* may be exploited as potential candidates for the treatment of cutaneous leishmaniasis. However, further studies are required to understand the components of the larval products and the mechanisms of inhibitory activities on leishmania parasites.

## Abbreviations

CL: Cutaneous leishmaniasis
ACL: Anthroponotic cutaneous leishmaniasis
AmB: Amphotericin B
ES: Excretion and secretion
AMPs: Anti-microbial peptides
SGLs: Salivary gland lysates
SPH-TUMS: School of Public Health, Tehran University of Medical Sciences
IPA: Isopropyl alcohol
SDS-PAGE: Sodium dodecyl sulfate-polyacrylamide gel electrophoresis
PBS: Phosphate buffered saline
BCA: Bicinchoninic acid
BSA: Bovine serum albumin
CRTSDL: Center for Research and Training in Skin Diseases and Leprosy
NNN: Novy-Macneal-Nicolle
FBS: Fetal bovine serum
RPMI: Roswell Park Memorial Institute
DMEM: Dulbecco’s Modified Eagle Medium
IP: Intraperitoneally
MTT: 3-(4.5-Dimethylthiazol-2-yl)-2,5-diphenyltetrazolium bromide
DMSO: Dimethyl sulfoxide
OD: Optical density
CC50: Cell cytotoxicity for 50% of cells
IC50: Inhibitory concentration for 50% of parasite
SI: Selectivity index
%I: Infection percentage
%DI: Decreased infection percentage
%V: Viability of amastigote percentage
%DV: Decreased viability of amastigote percentage
GEE: Generalized estimating equation
SVI: Survival index
PL: Parasite load

## Availability of data and materials

The dataset analyzed during the current study is available from the corresponding author on reasonable request.
