# Correction to: The leishmanicidal efect of *Lucilia sericata* larval saliva and hemolymph on in vitro *Leishmania tropica*

**DOI:** 10.1186/s13071-021-04649-x

**Published:** 2021-03-15

**Authors:** Sara Rahimi, Ali Khamesipour, Amir Ahmad Akhavan, Javad Rafnejad, Reza Ahmadkhaniha, Mahmood Bakhtiyari, Arshad Veysi, Kamran Akbarzadeh

**Affiliations:** 1 Department of Medical Entomology and Vector Control, School of Public Health, Tehran University of Medical Sciences, Tehran, Iran; 2 Center for Research and Training in Skin Diseases and Leprosy, Tehran University of Medical Sciences, Tehran, Iran; 3 Pharmaceutical Chemistry, Department of Human Ecology, School of Public Health, Tehran University of Medical Sciences, Tehran, Iran; 4 Department of Community Medicine and Epidemiology, School of Medicine Non-communicable Diseases Research Center Alborz, University of Medical Sciences, Karaj, Iran; 5 Zoonoses Research Center, Research Institute for Health Development, Kurdistan University of Medical sciences, Sanandaj, Iran

## Correction to: Parasites Vectors (2021) 14:40 10.1186/s13071-020-04543-y

Following publication of the original article [1], the authors flagged that the name of the author ‘Reza Ahmadkhaniha’ had been misspelled as ‘Reza Ahmadkhaniaha’.

The name has been corrected in the original article and the corrected name may be found in this correction article.

The authors apologize for any inconvenience caused.
